# Psychological Traits of Patients With Depression Comorbid With Chronic Pain: Are Complaint and Competitive Tendency Related to Pain?

**DOI:** 10.3389/fpsyt.2022.825422

**Published:** 2022-02-10

**Authors:** Koji Fujimoto, Masako Hosoi, Ryoko Katsuki, Toshio Matsushima, Keitaro Matsuo, Tomohiro Nakao, Nobuyuki Sudo, Takahiro A. Kato

**Affiliations:** ^1^Department of Psychosomatic Medicine, Graduate School of Medical Science, Kyushu University, Fukuoka, Japan; ^2^Department of Psychosomatic Medicine, Kyushu University Hospital, Fukuoka, Japan; ^3^Multidisciplinary Pain Center, Kyushu University Hospital, Fukuoka, Japan; ^4^Department of Neuropsychiatry, Graduate School of Medical Sciences, Kyushu University, Fukuoka, Japan

**Keywords:** depression, chronic pain, personality traits, modern-type depression, alexithymia, TACS-22, achievement motive

## Abstract

**Background:**

Modern-Type Depression (MTD) is a category of depression that has been studied mainly in Japan; however, no study has attempted to determine its relation to chronic pain.

**Aim:**

To determine possible associations between psychological traits related to MTD and the chronic pain of patients at psychiatric clinics.

**Method:**

Two hundred and twenty-one first time patients who visited the psychiatric clinic at a Japanese university medical center or an associated clinic were enrolled. The Hamilton Depression Rating Scale was used to measure depressive symptoms. The 22-item Tarumi's Modern-Type Depression Trait Scale (TACS-22), Achievement Motive, and 20-item Toronto Alexithymia Scale were used to assess psychological traits related to depression and chronic pain. The clinical diagnosis of each patient was confirmed by use of the Structured Clinical Interview for DSM-IV Axis I Disorders, administered by experienced specialists. The medians of the psychological traits identified were compared between patients with or without chronic pain. Analysis was also done of patients with Major Depressive Disorder (MDD).

**Result:**

Of the 221 patients, 139 had chronic pain. Patients with chronic pain had more severe depressive symptoms, Alexithymia, and high scores for the complaint trait of MTD. Seventy-three of the 221 patients met the criteria for MDD (53 had chronic pain). Patients with MDD comorbid with chronic pain had a higher competitive achievement score, severe depression, and difficulty identifying feelings.

**Conclusion:**

Complaint and competitive traits were shown to be related to chronic pain in psychiatric settings. Further study will allow us to design multidimensional approach for patients suffering from depression.

## Introduction

The global prevalence of depression is estimated at 3.76%, and the prevalence in Japan was reported to be 2.66% in 2019 (1). Similarly, chronic pain affects around 30.3% of the worldwide population (2) and 46.4% of Japanese older than 40 years (3). Depressed patients often complain of chronic pain, and vice versa (4, 5), and psychosocial factors are known to be common to depression and chronic pain (6, 7).

Alexithymia has been reported to be associated with both depression and chronic pain. In a recent meta-analysis, the degree of alexithymia was correlated with pain intensity, physical interference, depressive symptoms, and anxiety in a group of chronic pain patients (8). Economic factors and social isolation have also been reported to be prognostic of depression and chronic pain (9, 10).

Modern-type depression (MTD), an independent type of depression that is different from the conventional depression based on a melancholy temperament, has been widely reported in Japan (11–13). This condition is characterized by the occurrence of depressive symptoms mainly in a stressful workplace or school setting, then rapid disappearance once the patient leaves the stressful situation (14). A self-reporting questionnaire, the 22-item Tarumi's Modern-Type Depression Trait Scale (TACS-22), has been designed to assess the premorbid personality of patients with MTD, including three factors such as Avoidance of Social Roles, Complaint, and Low Self-Esteem (15). MTD is regarded as a gateway disorder to a type of pathological social withdrawal called hikikomori in Japan (11, 16–18).

Achievement motive (AM) is another instrument used in Japan to assess psychological traits related to depressive symptoms. It consists of two aspects of motivation: competitive and self-fulfillment. Those whose competitive achievement motive is high have been reported to have difficulty accepting social support and to have a tendency toward stronger depressive symptoms (19).

Studies have related the personality traits of MTD and AM to depressive symptoms, but their relation to chronic pain is unknown. Thus, to clarify which traits are related to chronic pain, we used the above inventories to investigate the interaction between depression and personality traits of patients with or without chronic pain. The patients were recruited from psychiatric clinics because we expected many of them to suffer from depression. The level of depression and confirmation of the diagnosis of the patient's base disease were assessed by semi-structured interviews done by experienced psychiatrists or psychologists.

## Methods

### Data Collection

Patients who first visited the Mood Disorder/Hikikomori Clinic in the Department of Neuropsychiatry at Kyushu University Hospital or affiliated psychiatric institutes between July 2018 and February 2021 and who gave written consent were enrolled. Patients referred from other psychiatric medical institutions due to refractory symptoms and suspected of having a mood disorder were included. Patients who could not complete a face-to-face semi-structured interview and patients for whom detailed information about their pain was not available were excluded, leaving the data of 221 patients available for analysis.

This study was approved by the ethics committee of Kyushu University, Fukuoka, Japan. Participants agreed to join in the study after being informed that their anonymity would be maintained and that participation was voluntary.

### Measures

The study was cross-sectional and done through the use of self-reporting questionnaires and a structured interview of all participants to examine psychological factors related to their depression and pain. The questionnaires were used to assess specific patient psychological traits, factors related to their disease, and clinical data including age, gender, location and duration of pain, socioeconomic status (smoking, alcohol consumption, marital status, education, employment etc.). Pain lasting more than 3 months was considered chronic. The following evaluations were done.

#### The Structured Clinical Interview for DSM-IV Axis I Disorders

The SCID (Japanese version) is a semi-structured psychiatric interview with established reliability that assesses if the patient has reached a diagnostic threshold according to the DSM-IV (20). All of the interviewers were psychiatrists or psychologists with extensive clinical experience and familiarity with SCID-I administration procedures.

#### The Hamilton Depression Rating Scale

The Hamilton Depression Rating Scale (HDRS) is a structured interview that assesses the severity of depression (21). Answers to the Japanese version of the HDRS were assessed by trained psychiatrists or psychologists.

#### Patient Health Questionaire-9

The PHQ-9, this screening questionnaire is based on the diagnostic criteria of DSM-IV and consists of nine questions on depression in the preceding 2 weeks. It was used for assessment of depression severity (22). Answers are on a scale of 0–3, giving a maximum score of 27. A score of 20 or greater indicates severe depression. It can be administered in a short time and can be used to assess depression in primary care settings. The previously validated Japanese version was used (23).

#### The 22-Item Tarumi's Modern-Type Depression Trait Scale

TACS-22 is a self-reporting questionnaire that assesses the premorbid personality traits of MTD (15). The questionnaire consists of 22 items to be answered on a 5-point scale from 0 to 4. It has three subcategories: Avoidance of Social Roles, Complaint, and Low Self-Esteem.

#### The 20-Item Toronto Alexithymia Scale

TAS-20 is a 20 item self-reporting questionnaire that assesses Alexithymia-related personality traits (24). It consists of three subcategories: Difficulty identifying feelings (DIF), Difficulty describing feelings (DDF), and Externally oriented thinking (EOT). The previously evaluated Japanese version was used (25).

#### Achievement Motive

AM is a self-reporting questionnaire that evaluates the strength of motivation to achieve a goal. It consists of 23 questions (26) and assesses two aspects: Self-fulfillment achievement motive and Competitive achievement motive (19).

### Statistics

For the analysis, patients with pain lasting more than 3 months were placed in a chronic pain group (CP). All met the diagnostic criteria for major depression, and this group is referred to as MCP. Patients without pain (NP) were placed in a control group. All met the diagnostic criteria for major depression, and the group is referred to as MNP. The median values of each psychological scale measured were compared by Wilcoxon-Mann-Whitney test. Statistical analysis was done with R version 4.1.1. *P*-values of < 0.05 were considered to be statistically significant, and *P*-values of < 0.1 were considered as marginally significant.

## Results

**Figure 1:**
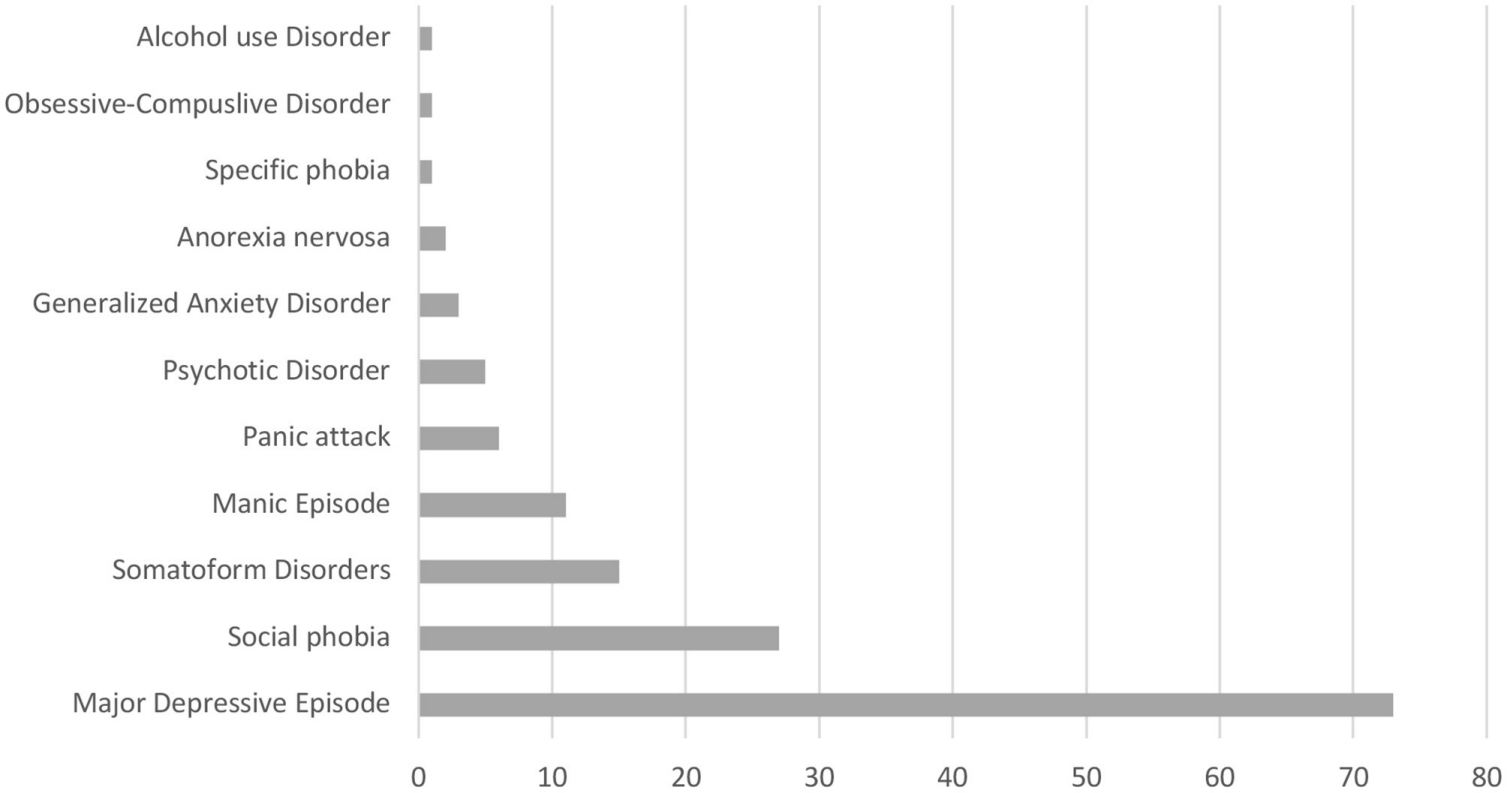
Frequency of psychiatric diagnosis based on DSM-IV.

The frequencies of the patients' primary disease for which the diagnostic threshold of the structured interview (SCID) was exceeded are summarized in Figure 1. Major depressive disorder (MDD, 73 patients) was the most common, followed by social phobia (27 patients) and somatoform disorders (15 patients). Twenty-eight patients met multiple diagnostic criteria. Descriptive statistics are shown in Table 1. Of the 221 patients enrolled, 139 had pain lasting more than 3 months (CP), 53 of whom had MDD (MCP).

**Table 1:** Demographic characteristics.

		**All (*n* = 221)**	**Major depression (*n* = 73)**	**No-Major depression (*n* = 148)**
With chronic pain (%)		139 (63%)	53 (73%)	86 (58%)
Age (mean ± SD years old)		33.8 ± 12.2	35.6 ± 12.0	32.9 ± 12.2
Sex (female)		117 (53%)	44 (60%)	73 (49%)
Current smoker (%)		41 (19%)	18 (24%)	23 (16%)
Alcohol drinking	Not at all (%)	132 (60%)	46 (62%)	86 (59%)
	<3 times a week (%)	65 (29%)	20 (27%)	45 (31%)
	More than 4 times a week (%)	24 (11%)	8 (11%)	16 (11%)
Martial status	Single (%)	130 (59%)	36 (49%)	94 (64%)
	Married (%)	69 (31%)	30 (41%)	38 (26%)
	Divorced or bereaved (%)	14 (6%)	5 (7%)	9 (6%)
Education	Secondary (%)	95 (43%)	27 (36%)	68 (46%)
	Higher vocational or Universities (%)	119 (54%)	34 (46%)	62 (42%)
Paid employment (%)		104 (47%)	38 (51%)	66(45%)
Antidepressant use (%)		31 (14%)	13 (18%)	18 (12%)
Living alone (%)		40 (18%)	14 (19%)	26 (18%)

A comparison of the median values of each psychological scale for CP (*n* = 139) and NP (*n* = 82) is shown in Table 2A. The median values of the severity of depression score on both PHQ-9 and HDRS were significantly higher in the CP group [PHQ9: 15 (CP)] vs. 10.5 (NP)/*p* = 0.00002, HDRS: 14 (CP) vs. 9 (NP), *p* = 0.0000001). The median TAS-20 score was also marginally higher in the CP group but was without significance [49 (CP) vs. 47 (NP)/*p* = 0.0532]. The median values were significantly high for Complaint of TACS-22 and DIF of TAS-20 [Complaint: 11 (CP) vs. 8.5 (NP)/*p* = 0.0199, DIF: 16 (CP) vs. 14 (NP)/*p* = 0.0128]. Although the median values for Low Self-esteem of TACS-22 were the same for CP and NP, the mean value was higher in CP [median: 16, mean 15.9 (CP) vs. 14.8 (NP)/*p* = 0.0315].

**Table 2A:** Comparison of medians (*n* = 221).

**Measurement**		**CP (patients with chronic pain)** **(*n* = 139)** **(SD)**	**NP (patients without chronic pain)** **(*n* = 82)** **(SD)**	***P***
HDRS		14 (7.1)^***^	9 (6.1)	<0.001
PHQ-9		15 (6.7)^***^	10.5 (7.0)	<0.001
TACS-22	Total	50 (12.3)	47 (11.6)	0.15
	Avoidance of social roles	23 (6.3)	23.5 (6.8)	0.95
	Low self-esteem	16 (4.5)^*^	16 (4.1)	0.03
	Complaint	11 (5.5)^*^	8.5 (4.6)	0.02
Achievement motive	Total	109 (17.3)	105 (18.6)	0.93
	Self-fulfillment	64 (11.2)	63 (11.5)	0.71
	Competitive	45 (10.3)	44 (10.9)	0.61
TAS-20	Total	49 (9.7)	47 (9.4)	0.05
	Difficulty identifying feelings	16 (6.1)^*^	14 (6.3)	0.01
	Difficulty describing feelings	12 (3.1)	12 (3.0)	0.68
	Externally oriented thinking	21 (4.1)	21 (3.8)	0.90

* p < 0.05, **p < 0.01,

*** *p < 0.001. CP, patients with chronic pain; NP, patients without chronic pain; SD, standard deviation; HDRS, Hamilton Depression Rating Scale; PHQ-9, Patient Health Questionaire-9; TACS-22, The 22-item Tarumi's Modern-Type Depression Trait Scale; TAS-20, the 20-item Toronto Alexithymia Scale*.

The median values of each psychological scale were compared for the patients of each group who reached the diagnostic threshold for major depression (Table 2B). Depressive symptoms were stronger in the MCP group (*n* = 53) than in the MNP group (*n* = 20) [HDRS: 19 (MCP) vs. 15 (MNP)/*p* = 0.0111]. The median TAS-20 score was also marginally higher in the MCP group, although it did not reach the level of significance [52 (MCP) vs. 48.5 (MNP)/*p* = 0.0678]. The median value of the MCP group was higher for the DIF of TAS-20 [19 (MCP) vs. 14 (MNP)/*p* = 0.00211]. In addition, the competitive achievement motive of the AM inventory was higher in the MCP group [45 (MCP) vs. 40 (MNP)/*p* = 0.0163].

**Table 2B:** Comparison of medians of the MDD Patients (*n* = 73).

**Measurement**		**MCP (MDD patients with chronic pain) (*n* = 53) (SD)**	**MNP (MDD patients without chronic pain) (*n* =20) (SD)**	***P***
HDRS		19 (6.3)^*^	15 (3.9)	0.01
PHQ-9		19 (3.7)	17 (5.3)	0.06
TACS-22	Total	52 (11.0)	52 (7.8)	0.50
	Avoidance of social roles	24 (5.9)	23.5 (5.8)	0.88
	Low self-esteem	18 (3.6)	17.5 (3.6)	0.77
	Complaint	12 (5.0)	9 (3.6)	0.11
Achievement motive	Total	109 (15.7)	102 (16.4)	0.06
	Self-fulfillment	64 (9.4)	61 (11.6)	0.26
	Competitive	45 (9.8)^*^	40 (8.8)	0.02
TAS-20	Total	52 (9.1)	48.5 (6.1)	0.07
	Difficulty identifying feelings	19 (5.3)^*^	14 (4.2)	0.002
	Difficulty describing feelings	13 (2.8)	13 (2.0)	0.46
	Externally oriented thinking	20 (4.1)	21 (3.3)	0.22

* p < 0.05, **p < 0.01, ***p < 0.001. MCP, MDD patients with chronic pain; MNP, MDD patients without chronic pain; SD, standard deviation; TACS-22, The 22-item Tarumi's Modern-Type Depression Trait Scale; TAS-20, the 20-item Toronto Alexithymia Scale.

## Discussion

In our study to clarify the psychological characteristics of psychiatric outpatients with or without pain, patients with pain lasting more than 3 months showed more depressive symptoms and a tendency toward Alexithymia. This tendency was also observed in patients who met the diagnostic criteria for major depression. For all patients analyzed, personality traits related to MTD were stronger in the group with chronic pain, but there was no significant difference when only patients with major depression were included. The competitive achievement motive “aiming to beat or overcome others to be appreciated by others or society” did not differ between patients with or without chronic pain, but in the group with major depression and chronic pain the score was higher than for those without chronic pain.

The results showing that depressive symptoms were stronger in the group with chronic pain are consistent with the results of previous studies (4). Our study was unique in that we focused on personality traits. No significant difference in the total score of TACS or AM was seen for patients with or without chronic pain, but there was a significant difference in some of the subscales. The complaint item of the TACS-22 is a factor related to relationships with others and responsibility, and the competitive achievement motive is a factor related to gaining an advantage in comparison with others. These are important factors related to the development of social isolation. This is consistent with the results of a previous study (6) showing that depression partially mediates the relation between social isolation and pain interference. Sadly, the COVID-19 pandemic has increased loneliness and caused more severe social isolation (27). Increases in the incidence and intensity of pain and the prevalence of chronic pain have been reported in Japan (28). With the increased social isolation due to the pandemic, pain may become more prevalent among depressed patients.

The clinical manifestations, response to pharmacotherapy or psychotherapy, staging, and functioning of patients with depression are widely varied, and the development of personalized treatment modalities is necessary (29). To optimize treatment, multidimensional evaluation of depression is crucial (30). MTD is an exciting new concept of depression (11, 13, 14). Our findings will contribute to the development of multidimensional evaluation regimens related to specific personality traits.

### Limitations

Because this was an exploratory pilot study, its findings are limited. First, we did not assess the cause of chronic pain because the study was done in a psychiatric clinical setting with patients whose chief complaints were not pain. The sample size of MDD patients was relatively small, which limited the statistical analysis and does not allow us to draw conclusive findings. In addition, we did not use multiple test correction to avoid the risk of false negatives: the purpose of this study was to do a wide-ranging analysis of psychological traits as an exploratory pilot study as the basis for future validation studies. Despite the small sample size, we have found useful, statistically significant data. With the psychological trait candidates we were able to identify, follow-up studies with greater sample size will be able to be done to verify our preliminary findings.

## Conclusion

We found that psychiatric patients with chronic pain tend to have MTD-related psychological traits, notably “complaint to others.” Interestingly, patients with MDD who have chronic pain have a stronger tendency to be motivated by competitive aims than those without chronic pain. Our findings show that a complaining and competitive personality are related to chronic pain and depression, both of which are related to maintaining healthy relationships with others. MTD is known to be related to severe social isolation, hikikomori (17, 31), and further studies need to be done to clarify the interactions between chronic pain, depression, MTD, and hikikomori.

## Data Availability Statement

The raw data supporting the conclusions of this article will be made available by the authors, without undue reservation.

## Ethics Statement

This study was approved by Ethics Committee of Kyushu University, Fukuoka, Japan. The patients/participants provided their written informed consent to participate in this study.

## Author Contributions

TK initially designed the study, oversaw data analysis, participated in data interpretation, and writing of the manuscript. RK participated in study design and statistical analyses. KF conducted the statistical analyses, the literature searches, and wrote the manuscript. MH, TM, and KM participated in data interpretation and writing of the manuscript. TN and NS reviewed the manuscript for important intellectual content. All authors contributed to the article and approved the submitted version.

## Funding

This work was partially supported by Grant-in-Aid for Scientific Research on KAKENHI - the Japan Society for the Promotion of Science (JP15K15431, JP16H03741, JP16H06403, JP18H04042, JP19K21591, and JP20H01773 to TK, and JP19H03752 to MH), The Japan Agency for Medical Research and Development (AMED) (JP19ek0610015 to MH and TK, and JP17dk0307047, JP19dk0307073, JP18dk0307075, and JP21wm0425010 to TK), and SENSHIN Medical Research Foundation (to TK). The funders had no role in study design, data collection and analysis, decision to publish, or preparation of the manuscript.

## Conflict of Interest

The authors declare that the research was conducted in the absence of any commercial or financial relationships that could be construed as a potential conflict of interest.

## Publisher's Note

All claims expressed in this article are solely those of the authors and do not necessarily represent those of their affiliated organizations, or those of the publisher, the editors and the reviewers. Any product that may be evaluated in this article, or claim that may be made by its manufacturer, is not guaranteed or endorsed by the publisher.

## References

[1] Abbafati C Abbas KM Abbasi-Kangevari M Abd-Allah F Abdelalim A Abdollahi M. Global burden of 369 diseases and injuries in 204 countries and territories, 1990–2019: a systematic analysis for the global burden of disease study 2019. Lancet. (2020) 396:1204–22. 10.1016/S0140-6736(20)30925-9 33069326 PMC7567026

[2] Elzahaf RA Tashani OA Unsworth BA Johnson MI. The prevalence of chronic pain with an analysis of countries with a human development index less than 0.9: a systematic review without meta-analysis. Curr Med Res Opin. (2012) 28:1221–9. 10.1185/03007995.2012.703132 22697274

[3] Anno K Shibata M Ninomiya T Iwaki R Kawata H Sawamoto R. Paternal and maternal bonding styles in childhood are associated with the prevalence of chronic pain in a general adult population: the hisayama study. BMC Psychiatry. (2015) 15:181. 10.1186/s12888-015-0574-y 26227149 PMC4520085

[4] Velly AM. Epidemiology of pain and relation to psychiatric disorders. Prog Neuropsychopharmacol Biol Psychiatry. (2018) 87:159–67. 10.1016/j.pnpbp.2017.05.012 28522289

[5] IsHak WW Wen RY Naghdechi L Vanle B Dang J Knosp M. Pain and depression: a systematic review. Harv Rev Psychiatry. (2018) 26:352–63. 10.1097/HRP.0000000000000198 30407234

[6] Johnston KJA Adams MJ Nicholl BI Ward J Strawbridge RJ Ferguson A. Genome-wide association study of multisite chronic pain in UK biobank. PLoS Genet. (2018) 15:502807. 10.1101/502807 31194737 PMC6592570

[7] Roughan WH Campos AI García-Marín LM Cuéllar-Partida G Lupton MK Hickie IB. Comorbid chronic pain and depression: shared risk factors and differential antidepressant effectiveness. Front Psychiatry. (2021) 12:1–13. 10.3389/fpsyt.2021.643609 33912086 PMC8072020

[8] Aaron R V Fisher EA Lumley MA Tonya M States U States U. Alexithymia in individuals with chronic pain and its relation to pain intensity, physical interference, depression, and anxiety: a systematic review and meta-analysis. Pain. (2019) 160:994–1006. 10.1097/j.pain.0000000000001487.Alexithymia 31009416 PMC6688175

[9] Fisher E Heathcote LC Simons LE Palermo TM. Assessment of pain anxiety, pain catastrophizing, and fear of pain in children and adolescents with chronic pain: a systematic review and meta-analysis. J Pediatr Psychol. (2018) 43:314–25. 10.1093/jpepsy/jsx103 29049813 PMC6927870

[10] Karayannis NV Baumann FI Sturgeon JA Melloh M Mackey SC. The impact of social isolation on pain interference : a longitudinal study. Ann Behav Med. (2019) 53:65–74. 10.1093/abm/kay017 29668841 PMC6301311

[11] Kato TA Shinfuku N Sartorius N Kanba S. Are Japan's hikikomori and depression in young people spreading abroad? Lancet. (2011) 378:1070. 10.1016/S0140-6736(11)61475-X 21924990

[12] Kato TA Kanba S. Boundless syndromes in modern society: an interconnected world producing novel psychopathology in the 21st century. Psychiatry Clin Neurosci. (2016) 70:1–2. 10.1111/pcn.12368 26781189

[13] Kato TA Hashimoto R Hayakawa K Kubo H Watabe M Teo AR. Multidimensional anatomy of “modern type depression” in Japan: a proposal for a different diagnostic approach to depression beyond the DSM-5. Psychiatry Clin Neurosci. (2016) 70:7–23. 10.1111/pcn.12360 26350304 PMC5560068

[14] Kato TA Kanba S. Modern-type depression as an “adjustment” disorder in Japan: the intersection of collectivistic society encounteringanindividualisticperformance-basedsystem. Am J Psychiatry. (2017) 174:1051–53. 10.1176/appi.ajp.2017.17010059 29088934

[15] Kato TA Katsuki R Kubo H Shimokawa N Sato-Kasai M Hayakawa K. Development and validation of the 22-item tarumi's modern-type depression trait scale: avoidance of social roles, complaint, and low self-esteem (TACS-22). Psychiatry Clin Neurosci. (2019) 73:448–57. 10.1111/pcn.12842 30900331 PMC6850625

[16] Kato TA Kanba S Teo AR. Hikikomori: experience in Japan and international relevance. World Psychiatry. (2018) 17:105–6. 10.1002/wps.20496 29352535 PMC5775123

[17] Kato TA Kanba S Teo AR. Hikikomori : multidimensional understanding, assessment, and future international perspectives. Psychiatry Clin Neurosci. (2019) 73:427–40. 10.1111/pcn.12895 31148350

[18] Kato TA Kanba S Teo AR. Defining pathological social withdrawal: proposed diagnostic criteria for hikikomori. World Psychiatry. (2020) 19:116–7. 10.1002/wps.20705 31922682 PMC6953582

[19] Horino M Mori K. The effects of achievement motivation on relationships between depression and social support. Japanese J Educ Psychol. (1991) 39:308–15.

[20] First MB Spitzer RL Gibbon M Williams JBW. Structured Clinical Interview for DSM-IV-TR Axis I Disorders, Research Version, Patient Edition. (SCID-I/P). New York, NY: Biometrics Research, New York State Psychiatric Institute (2002).

[21] Williams JBW. A structured interview guide for the hamilton depression rating scale. Arch Gen Psychiatry. (1988) 45:742–7. 3395203 10.1001/archpsyc.1988.01800320058007

[22] Spitzer RL Kroenke K Williams JBW. Validation and utility of a self-report version of PRIME-MD. Prim Care Companion J Clin Psychiatry. (2000) 2:31. 10.1001/jama.282.18.1737 10568646

[23] Muramatsu K Miyaoka H Kamijima K Muramatsu Y Yoshida M Otsubo T. The patient health questionnaire, Japanese version: validity according to the mini-international neuropsychiatric interview-plus. Psychol Rep. (2007) 101:952–60. 10.2466/pr0.101.3.952-960 18232454

[24] Bagby M Parker JDA Taylor GJ. The twenty-item toronto alexithymia scale-1. Item selection and cross-validation of the factor structure. J Psychosom Res. (1994) 38:23–32. 8126686 10.1016/0022-3999(94)90005-1

[25] Komaki G Maeda M Arimura T Nakata A Shinoda H Ogata I. The reliability and factorial validity of the japanese version of the 20-item toronto alexithymia scale(TAS-20). Japanese J Psychosom Med. (2003) 43:839–46. 10.15064/jjpm.43.12_83917707259

[26] Horino M. Analysis and reconsideration of the concept of achievement motive. Japan J Educ Psychol. (1987) 35:148–54. 26603910

[27] Kato TA Sartorius N Shinfuku N. Forced social isolation due to COVID-19 and consequent mental health problems: lessons from hikikomori. Psychiatry Clin Neurosci. (2020) 74:506–7. 10.1111/pcn.13112 32654336 PMC7404367

[28] Yamada K Wakaizumi K Tabuchi T. Loneliness, social isolation, and pain following the COVID - 19 outbreak : data from a nationwide internet survey in Japan. Sci Rep. (2021) 11:18643. 10.1038/s41598-021-97136-3 34545110 PMC8452720

[29] Maj M Stein DJ Parker G Zimmerman M Fava GA De Hert M. The clinical characterization of the adult patient with depression aimed at personalization of management. World Psychiatry. (2020) 19:269–93. 10.1002/wps.20771 32931110 PMC7491646

[30] Reynolds CF. Optimizing personalized management of depression: the importance of real-world contexts and the need for a new convergence paradigm in mental health. World Psychiatry. (2020) 19:266–8. 10.1002/wps.20770 32931119 PMC7491608

[31] Teo AR Nelson S Strange W Kubo H Katsuki R Kurahara K. Social withdrawal in major depressive disorder: a case-control study of hikikomori in japan. J Affect Disord. (2020) 274:1142–6. 10.1016/j.jad.2020.06.011 32663943

